# Experiences and Perceptions of Risk Among Australian Men Who Have Sex with Men Who Engage in Sexual Strangulation

**DOI:** 10.1007/s10508-025-03328-7

**Published:** 2026-03-23

**Authors:** Scotia P. Mullin, Rita Hardiman, Alastair J. Sloan, Kelsey Hegarty

**Affiliations:** The University of Melbourne, The Royal Women’s Hospital, 720 Swanston Street, Carlton, VIC 3053 Australia

**Keywords:** Sexual strangulation, Choking, Asphyxia, Sexual health, LGBTQ+ health, Sexual orientation

## Abstract

Sexual strangulation—commonly referred to as “choking”—is a prevalent sexual practice among young people and individuals within the LGBTQ+ community, most prevalent in men who have sex with men. The practice of sexual strangulation is a potentially hazardous behavior that can have numerous deleterious health consequences. In some circumstances, strangulation can result in loss of consciousness, neurological injury, and death. While the health consequences of external pressure on the neck, which restricts blood flow and respiration, are self-evident to medical communities, the same level of knowledge is less common in the broader community. The lack of knowledge around the risks of incorporating strangulation into sexual practices is a public health and sexual health concern. Therefore, it is important to understand the current trends surrounding the perception of risk and the experience of risk in communities where the activity is prevalent, such as men who have sex with men.

Through 23 semi-structured interviews, we identified four key themes surrounding the experiences and perceptions of risk among Australian men who have sex with men who engage in sexual strangulation. These include the influences on experience and perception of risk, erroneous estimations surrounding an individual's and partner's competence, a disregard for risk, and the prioritization of enjoyment, harm reduction, and minimization strategies. This article lends valuable insight into the experiences and perceptions of risk in men who have sex with men and engage in strangulation during sexual activity. The article provides suggestions and future implications for medicine and education.

## Introduction

In medical contexts, “choking” is defined as an internal blockage of the airway, whereas “strangulation” is defined as an external application of pressure to the neck that is sufficient to reduce or stop blood and/or air flow (Sauvageau & Boghossian, 2010). The terminology surrounding compression of the neck in sexual settings is highly varied. The public often refers to strangulation as “choking” or “sexual choking” (Conte et al., 2025; Herbenick et al., 2023a, 2023b), while in BDSM and kink communities, “breath control” or “breath play” can also be used (Schori et al., 2022; Sheff, 2021). Academic literature (depending on the author’s field of origin/field of publication) may use a wide variety of words, including “[non-fatal/fatal] strangulation” or “neck compression,” “choking,” “sexual strangulation,” or “sexual choking” (Doberentz et al., 2019; Gill et al., 2013; Herbenick et al., 2022a; Hou et al., 2023; Kim et al., 2023; Sharman et al., 2025a; Victoire et al., 2022). Other words that are used less often include “yoking” (to refer to neck compression using the forearm/s) or “external pressure to the neck” or “asphyxia” when discussing the phenomenon broadly. Within this study, we have elected for the term “sexual strangulation” when discussing the phenomenon in erotic contexts, as it highlights the erotic and sexual circumstances in which the activity occurs, while also aligning with medical and legal scholarship. When discussing the term more broadly, “strangulation” is used.

Strangulation can take two forms: (1) manual strangulation involving parts of one’s own body or the body of someone else, or (2) the use of ligatures (Sharman et al., 2025b). Strangulation can be used as an act of violence, usually in the context of intimate partner violence or sexual violence (Glass et al., 2008; Mullin & Hardiman, 2026). However, strangulation is also used during sex to heighten physiological, emotional, and psychological arousal (Herbenick et al., 2022a).

Sexual strangulation was historically categorized as a sexually deviant behavior along with homosexual activity, aggressive fellatio, masturbation, fisting, and other forms of BDSM and fetish sexual activity (Amaral et al., 2016; Coluccia et al., 2016; De Block & Adriaens, 2013; Singh, 1999). Engaging in asphyxiation during sexual activity was often thought to be uncommon. A study of 1,214 gay and bisexual men’s sexual behavior found that only 8.1% of individuals engaged in sexual asphyxiation (Grov et al., 2010). Within lesbian women, sexual asphyxiation was even less common (Tomassilli et al., 2009). However, recent research has noted that strangulation is prevalent, particularly in young people. A study by Herbenick et al. (2024) included 3,638 respondents, 113 of whom identified as gay men and 91 who identified as bisexual men. It was found that up to 50% of gay men and 31% of bisexual men had reported being “choked” during sex between 1 and 5 times during their life, and 19% of gay men and 18% of bisexual men reported being “choked” during sex more than five times. Such trends have been mirrored in two separate studies from Australia (Sharman et al., 2024, 2025a).

The increased commonality of sexual strangulation may be associated with pornographic consumption, media portrayals, and popular culture (Herbenick et al., 2023b). Sharman et al. (2024, 2025a) have also reported, in Australia, that individuals who identified as gay or bisexual are more likely to be “choked” than men who identified as heterosexual, suggesting a comparatively higher risk to the MSM community.

### Sexual Strangulation and Perception of Risk

While consensual strangulation during sex can be arousing (Herbenick et al., 2022b), it is associated with significant physiological, neurological, and psychological risks including hypoxia, loss of consciousness, stroke, skeletal and cartilaginous fractures, ocular injuries, cognitive impairment, mental ill health, cardiac arrest, and death (Commins et al., 2024; De Boos, 2019; Dunn et al., 2025; Hawley et al., 2001; Herbenick et al., 2021; Hou et al., 2023; Huibregtse et al., 2022; Marr & Bugeja, 2025; Parekh et al., 2024; Patch et al., 2018). Despite this, consideration of strangulation as a high-risk, potentially dangerous activity is not widely held among the general public (Sharman et al., 2025b). The use of the term “choking” rather than the term “strangulation” in popular discourse, coupled with its ubiquitous inclusion in pornography and social media, may obscure and diminish perceptions of risk (Douglas et al., 2024; Herbenick et al., 2022b; Sharman et al., 2025b; Wright et al., 2023). While there is some evidence that BDSM communities view sexual strangulation as “extreme” or harmful (Douglas et al., 2024), young people within the general population often view sexual strangulation positively and associate it with love, trust, and intimacy (Herbenick et al., 2022a, 2022b, 2023b, 2024b; Sharman et al., 2025a). The current literature is limited in assessing the views of individuals over 50 years old. There is little awareness of the magnitude of risks associated with this behavior (Herbenick et al., 2022b; Sharman et al., 2025b).

### Current Challenges

While consensual strangulation has been implicated in accidental injury and death during sexual activity (Mullin & Hardiman, 2026), it has also been utilized to disguise intimate partner violence, sexual violence, and homicide through the “rough sex defense” (Bows & Herring, 2020; Hou et al., 2023; Marr & Bugeja, 2025; Mullin & Hardiman, 2026; Petreca & Patch, 2025; Schori et al., 2022; Sowersby et al., 2022). This has been an issue for both heterosexual and MSM communities alike; however, much research has focused on the experiences of heterosexual women. To date, there has been only one qualitative study that addressed men’s experiences with sexual choking; however, only 3 of the 21 men in the study were sexual minority men (Herbenick et al., 2024b), and more research is needed to gauge medicolegal needs within the community.

The present study provides a focused qualitative exploration of the sexual choking experiences of men who have sex with men. The study aims to provide insight into the experiences and perceptions of risk among this group, as well as harm minimization and education strategies that may be used to augment sociosexual, general public, and medicolegal understandings of the phenomenon. Additionally, understanding of MSM community members’ perceptions of risk is important for considering the “rough sex defense” as well as future experiences of consensual and non-consensual strangulation.

### Interpretative Phenomenological Analysis (IPA)

IPA guided this study (Smith et al., 2009). Phenomenological analysis in various forms has been used to understand a wide variety of phenomena among men who have sex with men, including patient–clinician interactions (Semlyen et al., 2018), masculinity and masculine identities (Ravenhill & de Visser, 2018), deafness (Beese & Tasker, 2022), and HIV/AIDS (Flowers et al., 2011; Skinta et al., 2014). IPA enables authentic, participant-led research, valuing the participants’ own lived experiences and the meaning they have derived from those experiences.

## Method

### Participants

Individuals were recruited using a combination of social media and snowball sampling. This study was part of a larger project, “Experiences of Strangulation Amongst Men Who Have Sex with Men Research Project,” which explores both consensual and non-consensual experiences of strangulation among men who have sex with men in Australia. An online graphic poster was circulated on social media sites, with a preamble about the study and the first author. The graphic used both the words “choking” and “strangulation” within the poster to ensure the largest audience was captured.

To be eligible for the study, individuals had to identify as men who have sex with men, have been strangled/choked or have strangled/choked another, be over the age of 18 years, currently residing in Australia, and have access to a device that can connect to the internet such as a mobile phone, tablet, or computer. Participants were directed to an online expression of interest (EOI) form to help us determine eligibility.

While participants were offered the opportunity to conduct the interview face to face on the University of Melbourne’s Parkville Campus, all participants elected to conduct the interview online via Zoom.

### Measures and Procedure

Of the 47 individuals who filled out the EOI, 24 were not eligible to participate, did not wish to be interviewed, or were lost to attrition. Given wider discussion in academia surrounding potential false responses or “bots” (Bonnamy et al., 2025; Goodrich et al., 2023; Johnson et al., 2023), we used a variety of methods (including EOI completion time, email/IP addresses, geolocation results, time of day access, and inappropriate response to EOI questions), and follow-up email confirmation and clarification of study requirements to combat potential false responses. Additionally, financial reimbursement (Participants were reimbursed $50 (AUD) for their time and effort) was only disclosed after initial screening and email confirmation to reduce motivation for disingenuous responses.

Interviews were conducted via Zoom and ranged from 21 to 86 min, with an average length of 47 min. A semi-structured interview format was used with seven (7) major questions to be discussed (Supplementary Material 1). The semi-structured questions were used as part of a larger study of men who have sex with men’s experiences of strangulation, and therefore, not all questions are directly related to the research covered in this article.

The interviewer (SM) aimed to be continually curious using active listening, silence, and verbal prompts to create a conversational atmosphere, build rapport, and decrease perceived and actual power inequalities between the interviewer and the participant (Norlyk & Harder, 2010; Smith et al., 2009; Tarzia, 2021).

While a clear definition of strangulation was supplied to participants via the plain language statement and again at the commencement of the interview, to reflect the individual's lived experience after the initial questions, if they used the term “choking,” the interviewer adapted their language to include the term choking to reflect the participant's lived experience of the phenomenon.

### Data Analysis

We utilized a seven-step protocol (Smith & Nezza, 2022; Smith et al., 2009) to analyze the data. Table 1 explores this in more detail, also explaining how we approached each step.

**Table 1:** Interpretive phenomenological analysis approach

Steps	Purpose/aim	Steps taken
1. Data immersion	Develop a familiarity with the data and begin understanding the context in which the data has been produced	• Engaged with current literature (all authors) • Conducted and screened interviews • Transcribed by hand interviews • Reread transcripts and rewatched interview • Maintain a reflective journal • Interviews and transcription were conducted by SM as was the reflective journaling practices. A subset of transcripts were read (RH and KH) and a subset of interviews watched (RH) by other authors
2. Exploratory noting	Capture and consider initial observations and insights	• Mind mapping (SM and RH) • Annotated transcripts (SM) • Wrote a ‘case report’ for each participant (SM) • Transcript coding (SM, RH and KH led by SM) • Discussed with other authors (all authors) • Created initial experiential statements (SM)
3. Experiential statement development	Synthesize codes and experiential statements for each participant to create participant-level insights	• Reviewed and discussed trends, codes, and experiential statements with other authors and provide preliminary insights into emerging trends for each participant (all authors led by SM) • Created participant experiential statements (SM)
4. Searching for connections	Identify relationships between experiential statements for each participant	• Mind mapping (SM and RH) • Typed and printed off statements before arranging and clustering them on my office desk (SM and RH) • Discussed with other authors and explained thought process (all authors led by SM)
5. Identification and naming of personal experiential themes	Distill, identify, and name core participant experiential themes	• Refined clusters (SM and RH) • Counted the number of times similar thematic statements occurred throughout each participant’s transcript (SM) • Created Participant experiential themes through encompassing, combining, and examining thematic statements (SM, RH, KH) • Gave consideration to valuable quotes that encapsulate participant’s individual experience (all authors)
6. The next case	Maintain commitment to the idiographic approach	• Repeated Steps 1–5 for each of the participants independently while attempting to avoid influences from prior cases. SM did this for each of the 23 cases while authors RH and KH each reviewed a subset of participant transcripts
7. Identification and naming of group experiential themes	Identify shared experiences across the group and assess their commonality	• Conducted cross-case analysis (all authors led by SM) • Consideration of theme if more than 50% of participants discussed it as per research IPA research methodology recommendations (all authors led by SM) • Examined and count subthemes (SM and KH) • Reviewed (all authors), rewrite (SM), and edit (SM and KH) Group Experiential Themes • Group discussion (all authors led by SM) • Group Experiential Theme write up (SM), review, editing, and refinement (all authors)

#### Positionality and Reflexivity

Reflexivity and understanding one’s place in the world are integral parts of qualitative research. The authorship consists of two men (one gay, one heterosexual) and two women (both heterosexual), all of whom are of varying ages and Anglo-European descent. The authors possess a diverse range of academic and professional expertise, encompassing anthropology, anatomy, clinical medicine, general practice, public health, dentistry, and tissue engineering. Each of these characteristics has provided different perspectives of the latent meaning within the dataset, as well as an interpretation of the data and how it shapes our understanding of the participants and the phenomenon being discussed.

The research was led by a queer man who has lived experiences of sexual interactions with other men. The first author’s own queer identity and experiences were crucial in eliciting in-depth and uncensored information from the participants, facilitating immediate rapport and understanding between the participants and the interviewer. The author believes that his openness about being a gay man helped build rapport with participants and reveal commonalities; however, he also acknowledges that his anthropological and public health experience in interviewing has assisted in eliciting rich responses. It is also acknowledged that the shared queer identity may have led to assumptions about the researcher’s knowledge among participants, resulting in information being overlooked or not discussed. Considering this, all participants were also asked to clarify their experiences of choking/strangulation, how it occurred, with what implements, sexual practices, and other contextual information, as this was integral to the study.

## Results

### Participant Characteristics

A total of 23 men completed the semi-structured interview. Men were aged between 23 and 81 years. All but one (22 of 23) participant was assigned male at birth. All participants identified as male, men, transmasculine, or masculine presenting. Most individuals identified as gay (*n* = 19). Other individuals identified as bisexual (*n *= 3) or queer (*n* = 1). All individuals had reported engaging in sex with at least one individual who identified as a man at some point in their adult life. A breakdown of the basic demographic information is available in Table 2, along with participant pseudonyms.

**Table 2:** Participant demographic characteristics

Pseudonym	Age (years)	Gender	Sexual orientation	Sexual position preference
Xander	32	Man	Gay	Bottom
Finn	36	Man	Gay	Vers Top
Archie	33	Man	Gay	Vers
Ambrose	29	Man	Gay	Bottom
Peter	37	Man	Gay	Vers
Terrence	28	Man	Gay	Vers
Achilles	23	Man	Bisexual	Vers Bottom
Maven	32	Man	Gay	Bottom
Sam	39	Man	Bisexual	Vers
Oliver	62	Man	Gay	Bottom
Saxon	32	Man	Gay	Bottom
Clyde	63	Man	Gay	Vers
Oswald	81	Man	Gay	Bottom
Freddy	55	Man	Gay	Vers Bottom
James	45	Man	Gay	Vers Top
Larry	24	Man	Gay	Vers Bottom
Thane	43	Man	Gay	Vers Bottom
Elias	33	Man	Gay	Bottom
Quinn	49	Man	Gay	Vers
Chase	33	Man	Gay	Vers Top
Fox	36	Man	Gay	Vers Top
Ross	43	Non-binary person	Bisexual	Vers
Silver	24	Man	Queer	Side

Most participants had engaged in a sexual act where they were the recipient of sexual choking or strangulation, and over half of the participants discussed times that they were the choker or strangler. Most sexual activity involving strangulation discussed by participants occurred in the home; however, other places such as pubs/clubs/bars, cars, and sex-on-premises venues were mentioned. A full breakdown of contextual variables is noted in Table 3.

**Table 3:** Contextual variables mentioned by participants

Variable	Sub-variable	Count (n)
Sexual position		
	Top	0
	Vers Top	4
	Versatile	7
	Vers Bottom	4
	Bottom	7
	Side	1
Sexual orientation		
	Gay	19
	Bisexual	3
	Straight	0
	Queer	1
Strangulation/choking experience*		
	Strangler/Choker	13
	Being Strangled/Choked	21
Place of sexual encounter*		
	Pub/Club/Bar	4
	Car	2
	Outdoor Space	2
	Sex on Premises Venue	11
	Home	20
	Hotel	2

This count includes participants who mentioned the above contextual variables during their interview some participants may have experiences that they did not mention

^*^Individuals may have had more than one experience per variable

Four dominant themes were widely discussed by participants and assisted in understanding individual experiences and the perceptions of risk of sexual strangulation. These themes encompass knowledge acquisition, the influence of strangulation, erroneous estimations of risk perception, the balance between risk and the pursuit of pleasure, and harm reduction strategies (Table 4).

**Table 4:** Themes and subthemes discussed by participants

Theme	Sub-themes	Count (n)
Knowledge acquisition		23
	Porn	8
	Peers	5
	Partners	10
Estimations of risk		23
	Misjudgment	15
	Sufficient Knowledge	5
	Insufficient Knowledge	18
	Interpersonal Awareness	4
Doing it anyway		21
	Intrinsic Motivations	16
	Extrinsic Motivations	9
	Partner Motivations	7
Harm reduction		20
	Communication	18
	Assessment and Evaluation	11
	Intuition	10
	Techniques and Training	13

### Sexual Strangulation Situations

As discussed, “choking” can mean different things to different people. Within this sample, all 23 men explored experiences of choking that involved the pressure on the neck by a hand, hands, or the forearm. Individuals reported being strangled both face to face and from behind with “missionary” and “doggy style” being the most commonly described scenarios where choking occurred. Individuals used the words “choking,” “strangulation,” “asphyxia,” “breath play,” and “suffocation” to describe these experiences with “choking” and “strangulation” being the most common. Five participants also discussed “choking” and/or “strangulation” with ligature implements such as bondage/dog collars, rope, or belts.

A few individuals also described “choking” scenarios as internal obstructions to breathing, such as their throat being blocked by a penis in instances of fellatio or irrumation, termed by participants as “deepthroating,” “throat fucking,” “face fucking,” or blocking their own throat with a dildo or other sex toys. Some participants also noted that they were involved in other breath play activities, including autoerotic asphyxia, gas masks, ball gags, breath control, holding their breath, or having their mouth covered and/or nose pinched. This discussion was rare within the sample, with each being referenced thrice or less. (Please note: These instances were only included in further examination if an aspect of strangulation was also present during the acts.)

Most individuals were strangled when they were the receptive partner or “bottom” and most strangled others when they were the insertive partner or the “top,” during oral sex where they were the insertive partner, or during foreplay prior to sex. Only one individual noted being strangled when they were the insertive partner. These scenarios were more complex and dynamic in group sexual activity settings, and no clear patterns emerged.

### Peers, Partners, and Pornography: Knowledge Acquisition and Exchange Surrounding Sexual Strangulation

All participants widely discussed their initial entry into the world of sexual strangulation. All participants discussed that they had learned about sexual strangulation from their partners, their peers, or from the incorporation of strangulation in pornography (Table 4).

While multiple individuals reported learning from friends and peers, eight individuals noted that they learned about sexual strangulation from pornography; however, other individuals noted that pornography may have influenced their knowledge acquisition, as partners or friends who taught them may have learned from strangulation. Participants noted that engagement with pornographic materials had also shaped their perceptions surrounding the dangers (or perceived lack thereof) of sexual strangulation.

Fox, a gay man in his mid-thirties, contends that he is “of the porn generation,” highlighting that this coincides with individuals who are millennials. Fox, along with other participants, finds that “any sort of discussion about strangling in general can’t really get away from a discussion about porn,” highlighting the impact that this has had on men of this generation. Additional participants have discussed “getting ideas” or “trying things out” after viewing pornography, with one participant (Larry, gay man, 24) stating that “Sometimes you watch porn, you kind of take ideas from it and I talk to my sexual partners about it…” Many individuals have noted that the motivation to try such activities stems from their perceived arousing nature.

Most individuals referenced how they learned about strangulation during sex also influenced their perceptions of how dangerous the activity was, with individuals who learned from pornography perceiving the activity as less dangerous. However, some individuals also noted that they had learned about strangulation from their partner, who had learned about it from pornography; thus, the perceived risk was still low. Ross (bisexual person, 43) recounts their first experience of strangulation, which they found “quite enjoyable” even though there was only a “brief explanation” prior to engagement.

Ross’s experience of learning from their sexual partners was not uncommon. Ten men within the study discussed that their sexual partners were their prime informants regarding strangulation. In some circumstances, learning from sexual partners was disadvantageous as bias surrounding their profession, intelligence, and excitement in the activity influenced initial participation in strangulation and subsequent continued engagement. Xander presents these ideas as he shares his experiences surrounding the first time he had engaged in sexual strangulation:

“But like, He's a doctor, so I'm quite comfortable with like I was like “Okay, I'm just gonna go with it cause like, yeah.” And he was pretty good in sex... in the bedroom. So, I was like, “I'll just go with this”. Like, I'm sure he's not gonna do anything to hurt me. Right?”—Xander (Gay man, 32)

Xander recounts that while he did enjoy the experience, he learned a great deal by doing it, as “it wasn’t something we talked about beforehand.” The lack of explicit consent and the emphasis on learning by doing were not isolated to Xander but were discussed by numerous participants. Finn discussed a scenario in which he engaged in sexual activity with another man who “really wanted to be choked until he passed out…” While Finn (gay man, 36) was concerned about the potential health ramifications as well as his partner’s previous experience, noting that “he [the partner] hadn’t done it either,” he still engaged in the “hook-up.”

It was also common for individuals to learn about strangulation from their peers. James (gay man, 45) recounts a scenario in which he attended a camp for gay men, which involved a workshop on strangulation. At the same time, at a “camping thing” where he felt the instruction was less about pleasure and teaching strangulation was more, “A lesson in how to kill someone.” While James discusses his own opinions and actions in this scenario, it is also clear that others around him were also learning about the activity from peers and pseudo-professionals.

### On the Precipice: Erroneous Estimations of Risk

Participants often underestimated the risks of strangulation. While participants noted that their level of skill, knowledge, or interpersonal awareness minimized the risks of sexual strangulation, they often underestimated the risks to their health or the health of others. Additionally, some participants discussed that they were not prepared for the mental toll that participating in strangulation may have and the psychological ill effects that it may have on them or their relationship. The erroneous estimations of participants were often due to a misjudgment of risk, a lack of awareness of risk, varied interpersonal perceptions of risk, or an inability to make accurate risk assessments. Participants such as Ross (bisexual person, 43) discuss that they “believed that it was somewhat safer than I necessarily do now,” while other participants have noted witnessing people lose consciousness, reflecting that there are “risks that those people participating are not aware of” (Freddy, gay man, 55). Often, participants were unaware of the risks of serious injury or loss of consciousness and that continued education and engagement have altered the perception of some participants.

Participants also discussed times when they “passed out very slightly” (Peter, gay man, 37), or “not knowing my own strength” (Finn, gay man, 36), which led to their partner losing consciousness. Terrence (gay man, 28) reported feeling lightheaded, dizzy, and having strange feelings in their stomach during a group sexual encounter where he was strangled in a dark room, at a sex-on-premises venue, with a ligature. These examples were not isolated occurrences with most individuals reporting at least one similar experience.

Many participants reported that their perception of risk was lower than they initially thought, and their ability to estimate their own strength, recognize unconscious bias in others, and navigate safety concerns was also lower than they had anticipated.

Participant’s awareness of physical risks appears to have evolved through their experiences. For instance, when Finn’s partner loses consciousness without his awareness, he reflects on not realizing the lack of strength required to induce such a reaction. This realization brings a degree of caution and contemplation about the potential risks involved. While he recognizes these risks, he nonetheless continues to engage in and enjoy the act, indicating a nuanced balancing of risk and the pursuit of pleasure.

Finn, Peter, and Terrence’s experiences represent a trend among participants where participants were not aware of the ease with which an individual can lose consciousness from strangulation. Participants openly discussed how a “blood choke” (Chase, Gay man, 33), or how squeezing the sides of the neck was desirable, as opposed to pressing on the front of the neck. However, such information, coupled with an analysis of hand placement demonstrated by participants, highlighted the possibility of vascular occlusions, which could increase pressure in the brain, lead to rapid unconsciousness, or create ocular, facial, or intraoral petechiae. A few individuals within the study also reported such injuries that they were previously unaware of. In one scenario, while participating in sexual strangulation and asphyxial fellatio, Clyde relays that his partner “just went limp,” “stopped breathing,” and Clyde “had to give him CPR…”

### Doing It Anyway

While some participants had erroneous estimations of risk, others were acutely aware of the risks involved and decided to engage/continued to engage despite this awareness. Some participants discussed strangulation as a form of self-harm (Silver, Saxon, and Clyde). In contrast, one participant went as far as to label it an extreme behavior akin to amputation (James). However, most participants were somewhere in the middle, understanding strangulation as a complex balance of risk and reward. Participants openly discussed their cost–benefit analyses on how they believed strangulation could cause physical injury, stroke, trouble breathing, or risk of death. However, they chose to pursue the activity anyway due to the perceived benefits of psychological and physiological arousal.

Thane states after informing the interviewer that he has suffered a stroke in the past and is aware that sexual strangulation can cause strokes. Thane (gay man, 43) tempers this belief by talking about the positives of sexual strangulation and the benefits and enjoyment he has experienced, such as heightened arousal and connection with his partner. While his perception of risk is accurate, and Thane contends that “I did alright in science…” in Thane’s view, the positives he gains from strangulation outweigh the possible negative consequences. This trend is not unique to Thane but has been discussed by other participants who also have existing health issues, including mental health issues, epilepsy, and a history of injury from “rough sex” that involved strangulation.

Quinn (gay man, 49) is involved in the kink community and talks of his desire to be strangled to the point of unconsciousness. Even though Quinn is aware that “sexy as it is in the moment, we are dealing with something that is potentially dangerous and in worst case, life ending…” While Quinn is aware that serious harm may come to him, he believes that the enjoyment and benefits of his experiences outweigh any potential risks. Quinn discusses how engaging in strangulation, even though it is dangerous, is motivated by a complex flow of intrinsic and extrinsic factors. Individuals discuss that intrinsic factors of personal arousal and a desire for “adventurous,” “rough,” or “violent” sex outweigh the potential risks of strangulation and ideas of self-harm, stimulus seeking, or degradation leading to arousal. Such discussions are mirrored by other participants:

“I quite like the idea of, umm, people getting off to me, you know, being, being sort of, you know, used as a toy.”—Sam (Bisexual man, 39).

“I believe that it's probably an element of those and self-degradation, where I sort of go into sort of like a self-harm and new stimulus sort of space. And that probably leads to the arousal.”—Saxon (Gay man, 32).

These examples demonstrate that, like other risky activities, enjoyable yet risky activities are unlikely to be halted. Additionally, individuals discussed how strangulation can build trust and connection, and at times, this can be worth the risk of pushing boundaries. In addition to building trust in the relationship, many individuals also said that it enhanced their connection. Strangulation was seen as a way to connect and increase intimacy and meant that everyone had to be completely present during sex. Individuals talked about increased romantic and sexual connection, particularly by the recipient of the strangulation, who was also usually the bottom (the receptive partner) during sex. Individuals discussed that such dynamics where “the trust is immense” (Quinn, gay man, 49) and the feeling of powerlessness, domination, and that their partner is “the boss” (Ambrose, gay man, 29) and pushing their limits with participants noting that are integral to their motivations to engage in strangulation.

### “I’ve Got Limits”: Negotiating Harm Reduction and Risk Mitigation in Sexual Strangulation

Participants discussed four main harm reduction strategies: communication, assessment and evaluation of their partner’s knowledge and expertise, intuition, and gut instincts on whether to participate in strangulation with a certain partner, and practice of various skills involving strangulation.

Many participants had some perception of risk, whether that be the risk of injury or the risk that their partner might not respect their boundaries. While participants may have underestimated the risk that strangulation poses, particularly to their physical and mental health, many participants had harm reduction strategies, had engaged in negotiation about their boundaries or limits, or had demonstrated to their partner the ways that they would like the activity of strangulation to occur.

Freddy discusses his experiences of engaging in a master/slave dynamic in which he identifies as the “subjugate” or the “slave.” Freddy engages in a permanent power inequity, a dynamic which has been agreed upon and documented in a written contract. Freddy discusses that he has “been owned by” numerous partners. Freddy discusses that formal communications in the form of “written contract which says, you know what can and can’t be done and, and what are the rules and behaviors and protocols” are often used highlighting a form of communication which is not often discussed*.* Freddy highlights that “in one contract it was choking to the point of passing out was the limit” indicating a firm boundary for him and his partners.

While such strict dynamics and rigorous discussion were rare within the sample, many participants did engage in some level of casual conversation surrounding strangulation and their boundaries. Often, participants discussed “limits” or a “set of parameters” (Elias, gay man, 33). Most individuals often discuss consent in some way; however, it is rarely discussed in terms of active and enthusiastic consent, which is the current benchmark in Australia. Individuals often discussed using gut instincts, iterations of force, or have prior discussions to “gauge consent” (Archie, gay man, 33). Some individuals also discussed using their own hands to “guide” or “control” the level of force being used.

Some participants used mitigation strategies other than open discussion to engage in “safer” sexual strangulation. Oswald, an octogenarian, engages in strangulation during group sexual encounters with men and women. While Oswald is often engaging in group sex with men and women, and his primary partner is a woman, he still self-identifies as gay. He has transitioned to the strangler/choker rather than the recipient due to his age as “…being an older person, it might, I might go over the edge even, even sooner” (Oswald, gay man, 81)*.* Both he and his partner have expressed concerns over his welfare due to his age and perceived fragility. Oswald’s experiences highlight the potential risks and unique health concerns older adults who are sexually active might face, such as heightened physical vulnerability. Oswald acknowledges the importance of safety, indicating that he avoids bringing his partner to a point where they might pass out, which shows an awareness of risk.

Nevertheless, this does not translate to formal “safe words” or structured conversations about boundaries, suggesting an implicit, rather than explicit, approach to consent. Oswald and his primary partner’s relationship, defined by both closeness and physical separation (since they do not live together), is enhanced by shared experiences. Intimacy here is also associated with Oswald's sense of responsibility, as he feels he knows his partner’s limits well enough to keep the experience safe and enjoyable, ensuring his partner that “I’m not gonna take it too far to actually hurt you” which they feel comfortable with.

Another harm reduction strategy was assessing participants’ knowledge and skill with sexual strangulation. Prior to engaging, participants wished to assess their partners’ knowledge and previous experience with strangulation. Silver (queer man, 24) states that he aimed to “get an idea of the character and that knowledge” prior to engaging in strangulation in an attempt to avoid harm. Participants discussed knowledge of kink, strangulation technique, previous issues with blurred lines of consent, partner presentation, and discussions around previous sexual partners as a “risk assessment” tool to determine whether they wanted to engage in strangulation with a potential sexual partner. Similar to Chase’s statement “I am self-aware, or I-I feel emotionally aware enough to know that the difference between, umm, between someone causing harm and someone just having their fun…” Many participants echoed these sentiments in their interviews.

Participants also discussed multiple forms of choking/strangulation and presented preferences based on the feeling of danger, comfort level, and their perceived level of risk. Individuals discussed strangulation techniques, including one-handed, two-handed, yoking, and ligature suspension. Most participants discussed the preference for anterolateral compression over anterior compression. This is demonstrated by a quote from Chase, a gay 33-year-old man who prefers his partners “avoid the windpipe altogether and apply pressure to the part of the sides where the blood goes up” and assessed risk based on his level of physical comfort during strangulation experiences.

While individuals did discuss their harm minimization strategies with the researcher, those who were involved in the BDSM community did appear to be more explicit with their boundaries and harm minimization strategies. Some participants discussed extra precautions that they take during strangulation that they may not necessarily undertake in other BDSM-related activities. Participants discussed that they are involved in other activities such as spanking, watersports, fisting, or consensual non-consent. These participants often acknowledged that the precautions they take for strangulation or asphyxia-related activities are not always considered in other activities. James (Gay man, 45) discusses that while he participates in scat play, watersports, and fisting, he believes that “at the end of the day, you do not actually, you can’t actually do significant harm with that, you can’t do permanent neurological harm to somebody with scat…” Like James, other individuals who were hesitant to engage in strangulation often attempted to negotiate other activities, such as spanking (Fox, gay man, 36), to avoid strangulation and subsequent injuries or risks.

## Discussion

Using IPA, this study examined how men who have sex with men perceive and experience the risk of sexual strangulation that Sharman et al. (2024, 2025b) and others have noted is prevalent in Australia. Analysis of 23 interviews conducted with men who have sex with men yielded four major themes, including how individuals first learned of sexual strangulation, their varied (and often inaccurate) perceptions of risk, their choice to participate in sexual strangulation, their motivations for doing so, and participants’ ability to practice harm minimization.

The article builds on the work of previous research, which demonstrates varied perceptions of risk within the community (Conte et al., 2025). This study is congruent with current literature that the general public often underestimates the risk of strangulation and the impacts it can have on their health. These findings have been explored by Conte et al. (2025) who discuss that choking can be seen as “safe,” “fine,” or “good” and that many individuals within their sample believe that strangulation can be safe with the correct precautions such as consent, moderating “pressure,” or “intensity,” as well as trust and communication. Such beliefs were also mirrored within this study and demonstrate a notable tendency among individuals to misjudge numerous aspects of a sexual encounter. The balancing of risk and sexual pleasure has been documented in other aspects of MSM’s lives, including sex and HIV transmission (Prestage et al., 2012). Prestage et al. saw that gay men’s risk varied depending on contextual factors and personal factors and was qualitatively unique to everyone, which is in line with the experiences of men within this study, showing consistency in risk evaluation and mitigation regardless of the “risky” sexual activity.

Serious health ramifications from sexual strangulation recorded in the literature include bruising, difficulty swallowing, loss of consciousness, incontinence, petechial hemorrhages, and lower cognitive functioning (Commins et al., 2024; De Boos, 2019; Dunn et al., 2025; Hawley et al., 2001; Herbenick et al., 2021; Hou et al., 2023; Huibregtse et al., 2022; Parekh et al., 2024; Patch et al., 2018). This study presents no exception. However, in rare cases, skeletal fractures (De Boos, 2019; Marr & Bugeja, 2025), cartilaginous fractures (De Boos, 2019), and death have been recorded (Bauer et al., 2021; Schori et al., 2022). A recent study in Australia highlighted that, since 2000, 11 individuals have died during, or because of, sexual strangulation (Mullin & Hardiman, 2026). The authors contended that due to shame, stigma, and other factors, the number is most likely higher. Similar trends have also been seen in autoerotic activity (Byard, 1994; Byard & Winskog, 2012; Mullin et al., 2024), highlighting that both solo and interpersonal erotic activities involving asphyxia are dangerous.

While these situations may be rare in one-off strangulation engagements, many individuals do not simply engage once. Considering the evidence provided by this study, as well as the pathological, anthropological, and medical findings mentioned above, it is plausible to say that while the likelihood of serious injury is low in one-time participants, repeated exposure increases the likelihood of injuries, with repeated loss of consciousness being of grave concern.

Throughout the study, participants discussed different strategies to cease sexual strangulation or disengage, such as red-light systems, safe words, non-verbal cues, and agreed-upon time restraints of strangulation. However, other activities, such as fisting, aggressive fellatio, coprophilia, spanking, and other BDSM activities, did not receive the same restrictions or harm minimization strategies. The perception of risk in such activities may be due to subconscious absorption of societal perceptions of the danger of strangulation through the media (Herbenick et al., 2023b; Winton, 2008; Wright et al., 2022, 2023). These findings suggest that increased social media use and pornographic consumption in young people may present scenarios in which sexual strangulation can be normalized and the dangers minimized. This idea is discussed by Herbenick et al. (2023b), who found through an analysis of memes that the dangers of strangulation, including death, were commonly referenced and that death or loss of consciousness could occur from surpassing boundaries or underestimating the force and pressure involved in the sexual strangulation. However, while memes did reference such information, Herbenick et al. (2023b) have noted that such information often did not aim at harm reduction or education.

Most participants learned about strangulation from their sexual partners or their peers; however, some also learned directly from pornography. This aligns with existing research which illustrates that men who have sex with men utilize pornography to educate themselves on same-sex sexual behaviors and can impact how individuals perceive sexual activity, enjoyment, and consent, and thus, the medium requires attention (Brennan, 2022; Fritz & Bowling, 2023; Kendall, 2004; Kubicek et al., 2010; Morrison, 2004; Traeen & Nilson, 2006).

A significant knowledge deficit in safe sexual practices occurs when pornography is used for education (Klaassen & Peter, 2015; McCormack & Wignall, 2017) rather than enjoyment. Pornography exhibits a bias toward male sexual desires, damaging heteronormative scripts, unrealistic body image, and inaccurate portrayals of power, dominance, and submission (Brennan, 2022; Lofgren-Martenson & Mansson, 2010; Sun et al., 2016; Tsitsika et al., 2009). Due to these shortcomings, the sexual activities involved in pornographic sex may lead to inaccurate perceptions of sex between men in the real world. Such inaccuracies are potentially dangerous for perceptions surrounding strangulation and its level of safety and perceived risk, both of which have been expressed in interviews with participants within this study.

While some participants underestimated the risk of strangulation, some individuals understood the risks of the activity and chose to pursue it anyway. Participants validated their decisions by noting other risky activities that they partook in, such as casual sex with strangers, unprotected sex, drug use, alcohol use, and speeding while driving. While participants usually incorporated some harm reduction strategies, their acknowledgment that the activity was dangerous and partaking anyway was mainly motivated by physiological, psychological, and sexual arousal. Of particular interest is the complex interplay between the pursuit of risk and the incorporation of risk to heighten sexual pleasure.

Additionally, harm reduction strategies are not uncommon in risky behavior. However, the estimation of their success or likelihood of ending the engagement in such activity is variable at best. While participants presented scenarios involving safe words or safe signals, the consideration of the inability to demonstrate such behaviors during neck compression may not have been fully realized by the participants. This is mirrored by the early (and it must be noted in these authors’ opinions: unethical) work in the 1940s, which (Rossen et al., 1943) illustrated that when the neck is compressed, activities such as speaking or dropping an object (as is common in BDSM activities to indicate limits) were often unsuccessful. This highlights that while individuals may have “safe words” or “safe practices” in place, their ability to enact them may be compromised, leading to an inability to withdraw consent or stop the activity. This, coupled with inhibited motor or cognitive function from drugs or alcohol, may present scenarios in which harm reduction strategies are arbitrary. Alcohol can inhibit neurological functioning, decrease inhibitions, and impact decision-making and risk perception processes among MSM. While prevalence varies, alcohol or drug consumption before or during sex is not uncommon within MSM communities globally (Bustamante et al., 2025; Hardy et al., 2022; Herbenick et al., 2025; Hou et al., 2023; Murray et al., 2024; Nehl et al., 2012; Ristuccia et al., 2019; Rosinska et al., 2018; Santos et al., 2018; Xu et al., 2019). These factors, influenced by drug and alcohol consumption, are associated with higher risk sexual behaviors, which could include sexual strangulation. While harm reduction strategies are common, this article has demonstrated that individuals’ perceptions of risk regarding strangulation are heterogeneous, inconsistent, and often insufficiently articulated.

### Implications and Future Considerations

This article displays that the experiences and perceptions of MSM surrounding sexual strangulation are often underestimated, poorly understood, or disregarded. This, coupled with findings by Sharman et al. as well as Herbenick et al., highlights a concerning scenario in which injury from strangulation may go undetected or unmitigated and poses a potential for increases in preventable injury and/or death.

Insights from this research have revealed a clear need for enhanced health messaging in the future. Regarding this paper in its entirety, it is clear that the findings have potential implications for the future of sexual strangulation as a topic of academic research, public health intervention, policy, medicine, and dentistry.

### Education and Schooling

Kirby (1992) and Vivancos et al. (2013) reported that a large part of harm reduction messaging involved safer sex education in schools and was effective in decreasing sexually transmitted infections and “risky” sexual behavior. It was argued that school-based sexual health education involving the risks of strangulation may assist in countering problematic and dangerous views of sexual strangulation.

The age of consent within Australia is 16 or 17 years, depending on the state or territory, and there is substantial evidence that suggests most individuals are consuming pornography and engaging in sexual activities prior to this age (Bothe et al., 2019; Crabbe et al., 2024). Given these findings, the provision of foundational information surrounding the risks of strangulation during sexual activity in a school-based setting at this age may be warranted. The positive implications of sexual health education have been discussed by Vrankovich et al. (2025), who suggest that sexual health education is an important primary prevention method for sexual violence. While sexual strangulation and sexual violence can be tangentially linked, particularly when lines of consent are blurred, this is not always the case. However, with most parents supporting sexual health education in Australian schools, consideration of the incorporation of the risks of strangulation may be valuable. Such education may help combat unrealistic expectations of sexual strangulation and the misperceptions of risk.

While such education may prove beneficial, policy research, evaluation, and feasibility studies may need to be conducted prior to the implementation of such a program. While such in-depth discussion is beyond the scope of this paper, this may be a necessary next step not just for MSM but for all young people.

### BDSM/Kink

Most participants within the study identified strangulation as part of BDSM or kinky sex, and most identified as part of either of these communities. While strangulation during sex often occurred in the home, the majority of participants noted that they had also experienced sexual strangulation in spaces that were not the home, such as sex-on-premises venues, in cars, or at BDSM/kink parties.

Individuals also discussed that they had experienced or witnessed sexual strangulation during group sex settings. These scenarios present unique and novel information surrounding sexual strangulation. While such findings of MSM engaging in group sex are concordant with current literature (Birch et al., 2022; Callander et al., 2019; McInnes et al., 2009, 2011; Prestage et al., 2009), these findings present novel information regarding sexual strangulation dynamics.

Strangulation between two individuals within the home is often unwitnessed, and isolation is a leading contributing factor in autoerotic and erotic neck compression deaths (Byard, 1994; Mullin & Hardiman, 2026; Mullin et al., 2024). Thus, awareness of participation in less isolated areas, such as sex-on-premises venues, allows for a unique opportunity for public health messaging surrounding the risks of strangulation to increase public knowledge.

While we contend that there is no “safe” way to engage in sexual strangulation, it is unlikely that public health messaging will eliminate the practice. It would be beneficial to educate individuals within these spaces about the harms of strangulation and potential psychological and physiological responses that they may experience if they choose to participate.

In such areas where sex involving strangulation is occurring, it may be beneficial to train staff in basic first aid and to identify signs of an individual who has been strangled, such as petechiae on the face and eyes, difficulty swallowing, neck pain, and dizziness. While such injuries are not exclusive to strangulation and can occur from other sexual activities, information coupled with context and experience of working staff may prove valuable.

Additionally, due to the commonality of strangulation outlined by Sharman et al. (2025a), particularly in young Australians targeting kink or LGBTQ+ events that are not sexual in nature, may also be beneficial. Some individuals in the BDSM/kink community hold munches (personal correspondence) where they attend restaurants or picnics to meet like-minded people over food and drinks. Having educators on sexual health and strangulation attend such events with easily digestible information and the ability to have in-depth conversations may be desirable. One of the authors of this paper has also attended an LGBTQ+ makeup class where they were asked to present on strangulation in both consensual and non-consensual contexts prior to the makeup artist commencing the class. While the following list is not exhaustive, such presentations could also occur at drag brunches, drag shows, or at queer trivia nights.

### Medicolegal Implications

As strangulation in any context can be dangerous and risks are misunderstood by some participants in the activity, medical and dental professionals must be well placed to identify strangulation-related injuries and educate participants on the risks strangulation may pose to their well-being. Medical professionals such as physicians and nurses are well placed to identify signs of strangulation (Victoire et al., 2022).

The education of dentists on sexual strangulation, as well as non-consensual strangulation in various contexts, is essential. Due to expertise and training, dental professionals are well placed to identify intraoral injuries that may have been caused by strangulation, such as petechiae, tongue swelling, tongue hemorrhage, dysphagia, mucosal, and dental injuries (Gwinn et al., 2004). While such information has commonly been utilized in cases of abuse, the exact mechanism of injury is present whether the strangulation is consensual or not. Dentists’ training and close examination of the head and neck make them well placed to detect strangulation-related injuries during examination (Gwinn et al., 2004). By identifying such injuries and being open to discussion surrounding strangulation, dentists may be able to inform individuals of both broad risks and those directly related to their oral health. This multi-pronged approach would help capture individuals who have not recently discussed such items with their healthcare practitioner.

Additionally, the “rough sex” defense relies heavily on the idea that individuals were not aware of the risk that strangulation could pose to one’s health and that the act was consensual (Bows & Herring, 2020). This study, in line with other research, highlights that the perception of risk regarding strangulation is broadly misunderstood, and participants overestimate their ability to gauge their partner’s continued consent. There is also much discussion surrounding what an individual can consent to. Douglas et al. (2024) argue that the lines surrounding what constitutes informed consent during strangulation are difficult to ascertain both legally and for the individual themselves. Additionally, Douglas et al. discuss the thin line between sex and violence when strangulation is involved and highlight concern not only for individuals participating but also for the potential legal repercussions. This study highlights that knowledge around strangulation and poor perception of risk may lead to preventable deaths, but also creates a medicolegal loophole and validation of the rough sex defense. By providing further education, individuals are less likely to be able to maintain that ignorance, which can cause accidental death. While the rough sex defense is seldom used in Australian cases, such defenses or those that are similar still exist throughout the world. Understanding the perception of risk and the experiences of risk among specific populations may assist with more robust prosecution and defense in legal cases involving strangulation injury or death.

### Strengths and Limitations

To our knowledge, this is the first study to focus on the sexual choking experiences of men who have sex with men. The study aims to explore and explain individual perceptions of risk, harm reduction mechanisms, and individual decisions regarding such estimations. The article contributes to our current understanding of MSM sexual activity and contributes to growing discussions surrounding the inclusion and diversity of groups that were historically excluded from research. The study adds to existing literature by collecting qualitative data on Australian adults surrounding sexual strangulation/choking.

The article also incorporates an extensive age range from early twenties to early eighties and provides novel information on older adult sexual activities and BDSM involvement. The extensive age range gives voice to a large cohort of the MSM community and provides information about sexual activity and desire in older adults, which is still underexplored.

However, the study has its limitations. While inclusive sampling and recruitment were used to the best of the author’s ability, most participants within the study were white men, born in Australia. No participants within the study identified themselves as Aboriginal or Torres Strait Islander. Participants also presented with a wide range of sexual encounters in which strangulation occurred, including sex-on-premises venues, in group sexual activities, in casual sexual encounters, and with long-term partners. However, all individuals except three recounted that their strangulation experiences occurred exclusively with other individuals who identified as men. Given the increasing prevalence of bi + men and queer men, there does appear to be a distinct lack of such individuals within this sample. Additionally, there were few gender-diverse or transgender men within the sample. These limitations should be actively addressed in future research.

Another potential limitation was the choice to incorporate both the words “strangulation” and “choking” in the advertisement. While this may have led to an increased audience or capture rate, the extreme connotations associated with strangulation may have also inhibited some individuals from participating. While both were used, and strangulation was clearly defined in the plain language statement, there is the potential for self-selection bias.

### Conclusions

Sexual strangulation is a practice that has become commonplace within society, particularly among men who have sex with men (Herbenick et al., 2024b; Sharman et al., 2025a). Engagement in sexual strangulation occurred in a wide range of scenarios, including public spaces, sex-on-premises venues, at BDSM/Kink parties, and within the home and hotel rooms. We found that often individuals who were partaking in sexual strangulation were aware that the activity is associated with risk; however, most individuals did not accurately estimate the level of risk and overestimated their ability to mitigate or reduce such risks. Additionally, we found that MSM are likely to engage in harm minimization strategies to aid in navigating sexual strangulation. The voices of the 23 men within this study underscore the importance of continued safer sex education surrounding the potential risks of strangulation. Individuals who provide health care and other services, such as sex work or BDSM education, to men who have sex with men need to be aware of the risks of strangulation and adequately explain them to individuals who may wish to engage in the activity and are seeking additional information.
